# On generalization of refinement of Jensen’s inequality using Fink’s identity and Abel-Gontscharoff Green function

**DOI:** 10.1186/s13660-017-1521-x

**Published:** 2017-10-10

**Authors:** Tasadduq Niaz, Khuram Ali Khan, Josip Pečarić

**Affiliations:** 1 Department of Mathematics, University of Sargodha, Sargodha, 40100 Pakistan; 2 RUDN University, Miklukho-Maklaya str. 6, 117198, Moscow, Russia

**Keywords:** 26D07, 26D15, 26D20, 26D99, convex function, Jensen’s inequality, Fink’s identity, Abel-Gontscharoff interpolating polynomial, Green function for ‘two-point right focal’ problem

## Abstract

In this paper, we formulate new Abel-Gontscharoff type identities involving new Green functions for the ‘two-point right focal’ problem. We use Fink’s identity and a new Abel-Gontscharoff-type Green’s function for a ‘two-point right focal’ to generalize the refinement of Jensen’s inequality given in (Horváth and Pečarić in Math. Inequal. Appl. 14: 777-791, 2011) from convex function to higher order convex function. Also we formulate the monotonicity of the linear functional obtained from these identities using the recent theory of inequalities for *n*-convex function at a point. Further we give the bounds for the identities related to the generalization of the refinement of Jensen’s inequality using inequalities for the Cebyšev functional. Some results relating to the Grüss and Ostrowski-type inequalities are constructed.

## Introduction and preliminary results

Divided difference is a helpful tool when we are dealing with functions that have different degrees of smoothness. In [2], p.14, the divided difference is given as follows.

### Definition 1

Let *g* be a real valued function defined on $[\alpha,\beta ]$. For $r + 1$ distinct points $u_{0},u_{1}, \ldots,u_{r} \in [\alpha, \beta ]$, the *r*th order divided difference is defined recursively by

$$[u_{i};g] = g(u_{i})\quad i = 0,1, \ldots,r, $$

and

$$\begin{aligned}& [u_{0},u_{1}, \ldots,u_{r};g] \\& \quad = \frac{[u_{1},u_{2}, \ldots,u_{r};g] - [u_{0},u_{1}, \ldots,u_{r - 1};g]}{u_{r} - u_{0}}. \end{aligned}$$

This is equivalent to

$$[u_{0},u_{1}, \ldots,u_{r};g] = \sum _{j = 0}^{r} \frac{g(u_{j})}{w'(u _{j})}, $$

where $w(u) = \prod_{j = 0}^{r} (u - u_{j})$.

We can include the case when some or all points are the same. In this case

1 $$ [\underbrace{u,u, \ldots,u}_{l - \mathit{times}};f] = \frac{f^{(l - 1)}(u)}{(l - 1)!}, $$

where $f^{(l - 1)}$ is supposed to exist. The *r*-convex function is characterized by the *r*th order divided difference as follows (see [2], p.14).

### Definition 2

A function $g:[\alpha,\beta ] \to \mathbb{R} $ is called an *r*-convex function ($r \ge 0$) on $[\alpha,\beta ]$ if and only if

2 $$ [u_{0},u_{1}, \ldots,u_{r};g] \ge 0 $$

for all $(r + 1)$ distinct choices in $[\alpha,\beta ]$.

If the inequality is reversed then *g* is *r*-concave on $[\alpha,\beta ]$.

In [2], p.16, the following criterion is given to check the *n*-convexity of the function.

### Theorem 1.1

*If* $f^{(n)}$ *exists*, *then* *f* *is* *n*-*convex if and only if* $f^{(n)} \ge 0$.

In [1, 3] (see also [4], p.26), Horváth and Pečarić give a refinement of Jensen’s inequality for convex function. They define some essential notions to prove the refinement given as follows:

Let *X* be a set, and

$$\begin{aligned}& P(X): = \mbox{power set of }X, \\& \vert X\vert := \mbox{number of elements of }X, \\& \mathbb{N}:= \mbox{set of natural numbers with }0. \end{aligned}$$

Consider $q \ge 1$ and $r \ge 2$ be fixed integers. Define the functions

$$\begin{aligned}& F_{r,s}:\{ 1, \ldots,q\}^{r} \to \{ 1, \ldots,q \}^{r - 1}\quad 1 \le s \le r, \\& F_{r}:\{ 1, \ldots,q\}^{r} \to P \bigl( \{ 1, \ldots,q \}^{r - 1} \bigr), \end{aligned}$$

and

$$T_{r}:P \bigl( \{ 1, \ldots,q\}^{r} \bigr) \to P \bigl( \{ 1, \ldots,q\}^{r - 1} \bigr), $$

by

$$\begin{aligned}& F_{r,s}(i_{1}, \ldots,i_{r}): = (i_{1},i_{2}, \ldots,i_{s - 1},i _{s + 1}, \ldots,i_{r})\quad 1 \le s \le r, \\& F_{r}(i_{1}, \ldots,i_{r}): = \bigcup _{s = 1}^{r} \bigl\{ F_{r,s}(i_{1}, \ldots,i_{r}) \bigr\} , \end{aligned}$$

and

3 $$ T_{r}(I) = \textstyle\begin{cases} \phi, & I = \phi; \\ \bigcup_{(i_{1}, \ldots,i_{r}) \in I} F_{r}(i_{1}, \ldots,i_{r}), & I \ne \phi. \end{cases} $$

Next let the function

$$\alpha_{r,i}:\{ 1, \ldots,q\}^{r} \to \mathbb{N}\quad 1 \le i \le q $$

defined by

$$\alpha_{r,i}(i_{1}, \ldots,i_{r})\mbox{ is the number of occurrences of } i\mbox{ in the sequence }(i_{1}, \ldots,i_{r}). $$

For each $I \in P(\{ 1, \ldots,q\}^{r})$ let

$$\alpha_{I,i}: = \sum_{(i_{1}, \ldots,i_{r}) \in I} \alpha_{r,i}(i _{1}, \ldots,i_{r})\quad 1 \le i \le q. $$

($H_{1}$)
Let *n*, *m* be fixed positive integers such that $n \ge 1$, $m \ge 2$ and let $I_{m}$ be a subset of $\{ 1, \ldots,n \}^{m}$ such that
  4 $$ \alpha_{I_{m},i} \ge 1\quad 1 \le i \le n. $$

Introduce the sets $I_{l} \subset \{ 1, \ldots,n\}^{l}$ ($m - 1 \ge l \ge 1$) inductively by

$$I_{l - 1}: = T_{l}(I_{l})\quad m \ge l \ge 2. $$

Obviously the set $I_{1} = \{ 1, \ldots,n\}$ by ($H_{1}$) and this ensures that $\alpha_{I_{1},i} = 1$ ($1 \le i \le n$). From ($H_{1}$) we have $\alpha_{I_{l},i} \ge 1$ ($m - 1 \ge l \ge 1$, $1 \le i \le n$).

For $m \ge l \ge 2$, and, for any $(j_{1}, \ldots,j_{l - 1}) \in I _{l - 1}$, let

$$\mathsf{H} _{I_{l}}(j_{1}, \ldots,j_{l - 1}): = \bigl\{ \bigl((i_{1}, \ldots,i_{l}),k \bigr) \times \{ 1, \ldots,l\} \vert F_{l,k}(i_{1}, \ldots,i _{l}) = (j_{1}, \ldots,j_{l - 1}) \bigr\} . $$

With the help of these sets they define the functions $\eta_{I_{m},l}:I _{l} \to \mathbb{N}$ ($m \ge l \ge 1$) inductively by

$$\begin{aligned}& \eta_{I_{m},m}(i_{1}, \ldots,i_{m}): = 1 \quad (i_{1}, \ldots,i_{m}) \in I_{m}; \\& \eta_{I_{m},l - 1}(j_{1}, \ldots,j_{l - 1}): = \sum _{((i_{1}, \ldots,i_{l}),k) \in \mathrm{H}_{I_{l}}(j_{1}, \ldots,j_{l - 1})} \eta_{I_{m},l}(i_{1}, \ldots,i_{l}). \end{aligned}$$

They define some special expressions for $1 \le l \le m$, as follows:

5 $$\begin{aligned} \mathsf{A} _{m,l} =& \mathsf{A} _{m,l}(I_{m},x_{1}, \ldots,x_{n},p_{1}, \ldots,p_{n};f) \\ : =& \frac{(m - 1)!}{(l - 1)!} \sum_{(i_{1}, \ldots,i_{l}) \in I_{l}} \eta_{I_{m},l}(i_{1}, \ldots,i_{l}) \Biggl( \sum _{j = 1}^{l} \frac{p_{i_{j}}}{\alpha_{I_{m},i_{j}}} \Biggr) f \biggl( \frac{\sum_{j = 1}^{l} \frac{p_{i_{j}}}{\alpha_{I_{m},i_{j}}}x _{i_{j}}}{\sum_{j = 1}^{l} \frac{p_{i_{j}}}{\alpha_{I_{m},i_{j}}}} \biggr) \end{aligned}$$

and prove the following theorem.

### Theorem 1.2

*Assume* ($H_{1}$), *and let* $f:I \to \mathbb{R} $ *be a convex function where* $I \subset \mathbb{R} $ *is an interval*. *If* $x_{1}, \ldots,x_{n} \in I$ *and* $p_{1}, \ldots,p_{n}$ *are positive real numbers such that* $\sum_{i = 1}^{n} p_{i} = 1$, *then*

6 $$ f \Biggl( \sum_{s = 1}^{n} p_{s}x_{s} \Biggr) \le \mathsf{A} _{m,m} \le \mathsf{A} _{m,m - 1} \le \cdots \le \mathsf{A} _{m,2} \le \mathsf{A} _{m,1} = \sum_{s = 1} ^{n} p_{s}f ( x_{s} ). $$

In [5], A. M. Fink gave the following result.

If $f:[\alpha_{1},\alpha_{2}] \to \mathbb{R} $, where $[\alpha_{1},\alpha _{2}]$ is an interval, is a function such that $f^{(n - 1)}$ is absolutely continuous then the following identity holds:

7 $$\begin{aligned} f(z) =& \frac{n}{\alpha_{2} - \alpha_{1}} \int_{\alpha_{1}}^{\alpha_{2}} f(\zeta)\,d\zeta \\ &{} + \sum _{\lambda = 1}^{n - 1} \frac{n - \lambda }{ \lambda!} \biggl( \frac{f^{(\lambda - 1)}(\alpha_{2})(z - \alpha_{2})^{ \lambda } - f^{(\lambda - 1)}(\alpha_{1})(z - \alpha_{1})^{\lambda }}{ \alpha_{2} - \alpha_{1}} \biggr) \\ &{}+ \frac{1}{(n - 1)!(\alpha_{2} - \alpha_{1})} \int_{\alpha_{1}}^{\alpha _{2}} (z - \zeta)^{n - 1}F_{\alpha_{1}}^{\alpha_{2}}( \zeta,z)f^{(n)}( \zeta)\,d\zeta, \end{aligned}$$

where

8 $$ F_{\alpha_{1}}^{\alpha_{2}}(\zeta,z) = \textstyle\begin{cases} \zeta - \alpha_{1}, & \alpha_{1} \le \zeta \le z \le \alpha_{2}; \\ \zeta - \alpha_{2}, & \alpha_{1} \le z < \zeta \le \alpha_{2}. \end{cases} $$

The complete reference about Abel-Gontscharoff polynomial and theorem for ‘two-point right focal’ problem is given in [6].

The Abel-Gontscharoff polynomial for ‘two-point right focal’ interpolating polynomial for $n = 2$ can be given as

9 $$ f(z) = f(\alpha_{1}) + (z - \alpha_{1})f'( \alpha_{2}) + \int_{\alpha _{1}}^{\alpha_{2}} G_{1}(z,w)f''(w) \,dw, $$

where

10 $$ G_{1}(z,w) = \textstyle\begin{cases} \alpha_{1} - w, & \alpha_{1} \le w \le z; \\ \alpha_{1} - z, & z \le w \le \alpha_{2}. \end{cases} $$

In the next section, we will present our main results by introducing some new types of Green functions defined as

11 $$\begin{aligned}& G_{2}(z,w) = \textstyle\begin{cases} \alpha_{2} - z, & \alpha_{1} \le w \le z; \\ \alpha_{2} - w, & z \le w \le \alpha_{2}, \end{cases}\displaystyle \end{aligned}$$

12 $$\begin{aligned}& G_{3}(z,w) = \textstyle\begin{cases} z - \alpha_{1}, & \alpha_{1} \le w \le z; \\ w - \alpha_{1}, & z \le w \le \alpha_{2}, \end{cases}\displaystyle \end{aligned}$$

13 $$\begin{aligned}& G_{4}(z,w) = \textstyle\begin{cases} \alpha_{2} - w, & \alpha_{1} \le w \le z; \\ \alpha_{2} - z, & z \le w \le \alpha_{2}, \end{cases}\displaystyle \end{aligned}$$

which enables us to introduce some new Abel-Gontscharoff-type identities, stated in the following lemma.

### Lemma 1.3

*Let* $f:[\alpha_{1},\alpha_{2}] \to \mathbb{R}$ *be a twice differentiable function and* $G_{k}$ ($k = 2,3,4$) *be the ‘two*-*point right focal problem’*-*type Green functions defined by* (11)-(13). *Then the following identities hold*:

14 $$\begin{aligned}& f(z) = f(\alpha_{2}) - (\alpha_{2} - z)f'( \alpha_{1}) - \int_{\alpha _{1}}^{\alpha_{2}} G_{2}(z,w)f''(w) \,dw, \end{aligned}$$

15 $$\begin{aligned}& f(z) = f(\alpha_{2}) - (\alpha_{2} - \alpha_{1})f'( \alpha_{2}) + (z - \alpha_{1})f'( \alpha_{1}) + \int_{\alpha_{1}}^{\alpha_{2}} G_{3}(z,w)f''(w) \,dw, \end{aligned}$$

16 $$\begin{aligned}& f(z) = f(\alpha_{1}) + (\alpha_{2} - \alpha_{1})f'( \alpha_{1}) - ( \alpha_{2} - z)f'( \alpha_{2}) + \int_{\alpha_{1}}^{\alpha_{2}} G_{4}(z,w)f''(w) \,dw. \end{aligned}$$

### Proof

The proofs of these identities requires some simple integration scheme, therefore we just give the proof of (16) only as follows:

$$\begin{aligned} \int_{\alpha_{1}}^{\alpha_{2}} G_{4}(z,w)f''(w) \,dw =& \int_{\alpha_{1}} ^{z} G_{4}(z,w)f''(w) \,dw + \int_{z}^{\alpha_{2}} G_{4}(z,w)f''(w) \,dw \\ =& \int_{\alpha_{1}}^{z} (\alpha_{2} - w)f''(w)\,dw + \int_{z}^{\alpha _{2}} (\alpha_{2} - z)f''(w)\,dw \\ =& (\alpha_{2} - z)f'(z) - (\alpha_{2} - \alpha_{1})f'(\alpha_{1}) + f(z) - f( \alpha_{1}) + (\alpha_{2} - z)f'( \alpha_{2}) \\ &{}- (\alpha_{2} - z)f'(z) \\ =& (\alpha_{2} - z)f'(\alpha_{2}) - ( \alpha_{2} - \alpha_{1})f'(\alpha _{1}) - f(\alpha_{1}) + f(z). \end{aligned}$$

Simplifying we get the result (16).

We define the following functionals by taking the differences of refinement of Jensen’s inequality given in (6):

17 $$\begin{aligned}& \Theta_{1}(f) =\mathsf{A} _{m,r} - f \Biggl( \sum _{s = 1}^{n} p_{s}x_{s} \Biggr), \quad r = 1, \ldots,m, \end{aligned}$$

18 $$\begin{aligned}& \Theta_{2}(f) = \mathsf{A} _{m,r} - \mathsf{A} _{m,k},\quad 1 \le r < k \le m. \end{aligned}$$

Under the assumptions of Theorem 1.2, we have

19 $$ \Theta_{i}(f) \ge 0,\quad i = 1,2. $$

Inequalities (19) are reversed if *f* is concave on *I*. □

## Main results

### Theorem 2.1

*Assume* ($H_{1}$), *and let* $f:I = [\alpha_{1},\alpha_{2}] \to \mathbb{R} $ *be a function such that for* $m \ge 3$ (*an integer*) $f^{(m - 1)}$ *is absolutely continuous*. *Also*, *let* $x_{1}, \ldots,x_{n} \in I$, $p_{1}, \ldots,p_{n}$, *be positive real numbers such that* $\sum_{i = 1}^{n} p_{i} = 1$. *Assume that* $F_{\alpha_{1}} ^{\alpha_{2}}$, $G_{k}$ ($k = 1,2,3,4$) *and* $\Theta_{i}$ ($i = 1,2$) *are the same as defined in* (8), (10)-(13) *and* (17)-(18), *respectively*. *Then*:

(i) *For* $k = 1,3,4$ *we have the following identities*:
  20 $$\begin{aligned} \Theta_{i}(f) =& (m - 2) \biggl( \frac{f'(\alpha_{2}) - f'(\alpha_{1})}{ \alpha_{2} - \alpha_{1}} \biggr) \int_{\alpha_{1}}^{\alpha_{2}} \Theta _{i} \bigl(G_{k}( \cdot,w) \bigr)\,dw \\ &{} + \frac{1}{\alpha_{2} - \alpha_{1}} \int_{\alpha_{1}}^{\alpha_{2}} \Theta_{i} \bigl(G_{k}( \cdot,w) \bigr) \\ &{} \times \sum_{\lambda = 1}^{m - 3} \biggl( \frac{m - 2 - \lambda }{ \lambda!} \biggr) \bigl( f^{(\lambda + 1)}(\alpha_{2}) (w - \alpha_{2})^{ \lambda } - f^{(\lambda + 1)}(\alpha_{1}) (w - \alpha_{1})^{\lambda } \bigr)\,dw \\ &{}+ \frac{1}{(m - 3)!(\alpha_{2} - \alpha_{1})} \int_{\alpha_{1}}^{\alpha _{2}} f^{(m)}(\zeta) \\ &{}\times \biggl( \int_{\alpha_{1}}^{\alpha_{2}} \Theta_{i} \bigl(G _{k}( \cdot,w) \bigr) (w - \zeta)^{m - 3}F_{\alpha_{1}}^{\alpha_{2}}( \zeta,w)\,dw \biggr)\,d\zeta, \quad i = 1,2. \end{aligned}$$
(ii) *For* $k = 2$ *we have*
  21 $$\begin{aligned} \Theta_{i}(f) =& ( - 1) (m - 2) \biggl( \frac{f'(\alpha_{2}) - f'(\alpha _{1})}{\alpha_{2} - \alpha_{1}} \biggr) \int_{\alpha_{1}}^{\alpha_{2}} \Theta_{i} \bigl(G_{2}( \cdot,w) \bigr)\,dw \\ &{}+ \frac{( - 1)}{\alpha_{2} - \alpha _{1}} \int_{\alpha_{1}}^{\alpha_{2}} \Theta_{i} \bigl(G_{2}( \cdot,w) \bigr) \\ &{}\times \sum_{\lambda = 1}^{m - 3} \biggl( \frac{m - 2 - \lambda }{ \lambda!} \biggr) \bigl( f^{(\lambda + 1)}(\alpha_{2}) (w - \alpha_{2})^{ \lambda } - f^{(\lambda + 1)}(\alpha_{1}) (w - \alpha_{1})^{\lambda } \bigr)\,dw \\ &{}+ \frac{( - 1)}{(m - 3)!(\alpha_{2} - \alpha_{1})} \int_{\alpha_{1}} ^{\alpha_{2}} f^{(m)}(\zeta) \\ &{}\times \biggl( \int_{\alpha_{1}}^{\alpha_{2}} \Theta_{i} \bigl(G_{2}( \cdot,w) \bigr) (w - \zeta)^{m - 3}F_{\alpha_{1}}^{\alpha _{2}}( \zeta,w)\,dw \biggr)\,d\zeta,\quad i = 1,2. \end{aligned}$$

### Proof

(i) Using Abel-Gontsharoff-type identities (9), (15), (16) in $\Theta_{i}(f)$, $i = 1,2$, and using properties of $\Theta_{i}(f)$, we get

22 $$ \Theta_{i}(f) = \int_{\alpha_{1}}^{\alpha_{2}} \Theta_{i} \bigl(G_{k}( \cdot,w) \bigr)f''(w)\,dw,\quad i = 1,2. $$

From identity (7), we get

23 $$\begin{aligned} f^{\prime\prime }(w) =& (m - 2) \biggl( \frac{f'(\alpha_{2}) - f'(\alpha_{1})}{ \alpha_{2} - \alpha_{1}} \biggr) \\ &{}+ \sum_{\lambda = 1}^{m - 3} \biggl( \frac{m - 2 - \lambda }{\lambda!} \biggr) \biggl( \frac{f^{(\lambda)}( \alpha_{2})(w - \alpha_{2})^{\lambda - 1} - f^{(\lambda)}(\alpha_{2})(w - \alpha_{2})^{\lambda - 1}}{\alpha_{2} - \alpha_{1}} \biggr) \\ &{}+ \frac{1}{(m - 3)!(\alpha_{2} - \alpha_{1})} \int_{\alpha_{1}}^{\alpha _{2}} (w - \zeta)^{m - 3}F_{\alpha_{1}}^{\alpha_{2}}( \zeta,w)f^{(m)}( \zeta)\,d\zeta. \end{aligned}$$

Using (22) and (23) and applying Fubini’s theorem we get the result (20) for $k = 1,3,4$.

(ii) Substituting Abel-Gontschroff-type inequality (14) in $\Theta_{i}(f)$, $i = 1,2$, and following similar steps to (i), we get (21). □

### Theorem 2.2

*Assume* ($H_{1}$), *and let* $f:I = [\alpha_{1},\alpha_{2}] \to \mathbb{R} $ *be a function such that for* $m \ge 3$ (*an integer*) $f^{(m - 1)}$ *is absolutely continuous*. *Also*, *let* $x_{1}, \ldots,x_{n} \in I$, $p_{1}, \ldots,p_{n}$ *are positive real numbers such that* $\sum_{i = 1}^{n} p_{i} = 1$. *Assume that* $F_{\alpha_{1}} ^{\alpha_{2}}$, $G_{k}$ ($k = 1,2,3,4$) *and* $\Theta_{i}$ ($i = 1,2$) *are the same as defined in* (8), (10)-(13) *and* (17)-(18), *respectively*. *For* $m \ge 3$ *assume that*

24 $$ \int_{\alpha_{1}}^{\alpha_{2}} \Theta_{i} \bigl( G_{k}( \cdot,\zeta) \bigr) (w - \zeta)^{m - 3}F_{\alpha_{1}}^{\alpha_{2}}( \zeta,w)\,dw \ge 0,\quad \zeta \in [\alpha_{1},\alpha_{2}], $$

*for* $k = 1,3,4$. *If* *f* *is an* *m*-*convex function*, *then*

(i) *For* $k = 1,3,4$, *the following holds*:
  25 $$\begin{aligned} \Theta_{i}(f) \ge& (m - 2) \biggl( \frac{f'(\alpha_{2}) - f'(\alpha_{1})}{ \alpha_{2} - \alpha_{1}} \biggr) \int_{\alpha_{1}}^{\alpha_{2}} \Theta _{i} \bigl(G_{k}( \cdot,w) \bigr)\,dw \\ &{}+ \frac{1}{\alpha_{2} - \alpha_{1}} \int_{\alpha_{1}}^{\alpha_{2}} \Theta_{i} \bigl(G_{k}( \cdot,w) \bigr) \\ &{}\times \sum_{\lambda = 1}^{m - 3} \biggl( \frac{m - 2 - \lambda }{ \lambda!} \biggr) \bigl( f^{(\lambda + 1)}(\alpha_{2}) (w - \alpha_{2})^{ \lambda } \\ &{}- f^{(\lambda + 1)}(\alpha_{1}) (w - \alpha_{1})^{\lambda } \bigr)\,dw, \quad i = 1,2. \end{aligned}$$
(ii) *For* $k = 2$, *we have*
  26 $$\begin{aligned} \Theta_{i}(f) \le& ( - 1) (m - 2) \biggl( \frac{f'(\alpha_{2}) - f'(\alpha _{1})}{\alpha_{2} - \alpha_{1}} \biggr) \int_{\alpha_{1}}^{\alpha_{2}} \Theta_{i} \bigl(G_{2}( \cdot,w) \bigr)\,dw \\ &{} + \frac{( - 1)}{\alpha_{2} - \alpha _{1}} \int_{\alpha_{1}}^{\alpha_{2}} \Theta_{i} \bigl(G_{2}( \cdot,w) \bigr) \\ &{}\times \sum_{\lambda = 1}^{m - 3} \biggl( \frac{m - 2 - \lambda }{ \lambda!} \biggr) \bigl( f^{(\lambda + 1)}(\alpha_{2}) (w - \alpha_{2})^{ \lambda } \\ &{}- f^{(\lambda + 1)}(\alpha_{1}) (w - \alpha_{1})^{\lambda } \bigr)\,dw, \quad i = 1,2. \end{aligned}$$

### Proof

(i) Since $f^{(m - 1)}$ is absolutely continuous on $[\alpha_{1},\alpha_{2}]$, $f^{(m)}$ exists almost everywhere. Also, by Theorem 1.1, we have $f^{(m)}(\zeta) \ge 0$ for a.e. on $[\alpha_{1}, \alpha_{2}]$. So, applying Theorem 2.1, we obtain (25).

(ii) Similar to (i). □

## Bounds for identities related to generalization of refinement of Jensen’s inequality

For two Lebesgue integrable functions $f_{1},f_{2}:[\alpha_{1},\alpha _{2}] \to \mathbb{R} $, we consider the Čebyšev functional

27 $$\begin{aligned} \Omega (f_{1},f_{2}) =& \frac{1}{\alpha_{2} - \alpha_{1}} \int_{\alpha _{1}}^{\alpha_{2}} f_{1}(t)f_{2}(t) \,dt \\ &{}- \frac{1}{\alpha_{2} - \alpha _{1}} \int_{\alpha_{1}}^{\alpha_{2}} f_{1}(t)\,dt \cdot \frac{1}{\alpha _{2} - \alpha_{1}} \int_{\alpha_{1}}^{\alpha_{2}} f_{2}(t)\,dt, \end{aligned}$$

where the integrals are assumed to exist.

In [7], the following theorems are given.

### Theorem 3.1

*Let* $f_{1}:[\alpha_{1},\alpha_{2}] \to \mathbb{R} $ *be a Lebesgue integrable function and* $f_{2}:[ \alpha_{1},\alpha_{2}] \to \mathbb{R} $ *be an absolutely continuous function with* $(\cdot - \alpha_{1})(\cdot - \alpha_{2})[f_{2}']^{2} \in L[ \alpha_{1},\alpha_{2}]$. *Then we have the inequality*

28 $$ \bigl\vert \Omega (f_{1},f_{2}) \bigr\vert \le \frac{1}{\sqrt{2}} \bigl[\Omega (f_{1},f_{1}) \bigr]^{ \frac{1}{2}}\frac{1}{\sqrt{\beta - \alpha }} \biggl( \int_{\alpha _{1}}^{\alpha_{2}} (x - \alpha_{1}) ( \alpha_{2} - x) \bigl[f_{2}'(x) \bigr]^{2}\,dx \biggr) ^{\frac{1}{2}}. $$

The constant $\frac{1}{\sqrt{2}} $ in (28) is the best possible.

### Theorem 3.2

*Let* $f_{1}:[\alpha_{1},\alpha_{2}] \to \mathbb{R} $ *be an absolutely continuous with* $f_{1}' \in L _{\infty } [\alpha_{1},\alpha_{2}]$ *and let* $f_{2}:[\alpha _{1},\alpha_{2}] \to \mathbb{R} $ *is monotonic non*-*decreasing on* $[ \alpha_{1},\alpha_{2}]$. *Then we have the inequality*

29 $$ \bigl\vert \Omega (f_{1},f_{2}) \bigr\vert \le \frac{1}{2(\alpha_{2} - \alpha_{1})}\Vert f_{1}'\Vert _{\infty } \int_{\alpha_{1}}^{\alpha_{2}} (x - \alpha_{1}) ( \alpha_{2} - x) \bigl[f_{2}'(x) \bigr]^{2}\,df_{2}(x). $$

The constant $\frac{1}{2}$ in (29) is the best possible.

Now we consider Theorem 3.1 and Theorem 3.2 to generalize results given in previous section. Let us first denote for $\zeta \in [\alpha_{1}, \alpha_{2}]$

30 $$\begin{aligned}& \mathsf{K} (\zeta) = \int_{\alpha_{1}}^{\alpha_{2}} \Theta_{i}\bigl(G_{k}( \cdot,w)\bigr) (w - \zeta)^{n - 3}F_{\alpha_{1}}^{\alpha_{2}}(\zeta,w)\,dw, \quad k = 1,3,4, \end{aligned}$$

31 $$\begin{aligned}& \hat{\mathsf{K} }(\zeta) = ( - 1) \int_{\alpha_{1}}^{\alpha_{2}} \Theta _{i} \bigl(G_{2}( \cdot,w) \bigr) (w - \zeta)^{n - 3}F_{\alpha_{1}}^{\alpha_{2}}( \zeta,w)\,dw, \quad i = 1,2. \end{aligned}$$

### Theorem 3.3

*Assume* ($H_{1}$), *let* $m \ge 3$ *be an integer*, *and* $f:[\alpha_{1},\alpha_{2}] \to \mathbb{R} $ *be such that* $f^{(m)}$ *is absolutely continuous with* $(\cdot - \alpha_{1})(\alpha_{2} -\cdot)[f^{(m + 1)}]^{2} \in L[\alpha_{1},\alpha_{2}]$. *Let* $p_{1}, \ldots,p_{n}$ *be positive real numbers such that* $\sum_{i = 1}^{n} p_{i} = 1$. *Also*, *assume* $F_{\alpha_{1}}^{\alpha_{2}}$ *and* $\Theta_{i}$ ($i = 1,2$) *are the same as defined in* (8) *and* (17)-(18), *respectively*. *Then*:

(i) *For* $G_{k}( \cdot,w)$ ($k = 1,3,4$) *as defined in* (10), (12) *and* (13), *respectively*, *we have*
  32 $$\begin{aligned} \Theta_{i}(f) =& (m - 2) \biggl( \frac{f'(\alpha_{2}) - f'(\alpha_{1})}{ \alpha_{2} - \alpha_{1}} \biggr) \int_{\alpha_{1}}^{\alpha_{2}} \Theta _{i} \bigl(G_{k}( \cdot,w) \bigr)\,dw \\ &{}+ \frac{1}{\alpha_{2} - \alpha_{1}} \int_{\alpha_{1}}^{\alpha_{2}} \Theta_{i} \bigl(G_{k}( \cdot,w) \bigr) \\ &{}\times \sum_{\lambda = 1}^{m - 3} \biggl( \frac{m - 2 - \lambda }{ \lambda!} \biggr) \bigl( f^{(\lambda + 1)}(\alpha_{2}) (w - \alpha_{2})^{ \lambda } - f^{(\lambda + 1)}(\alpha_{1}) (w - \alpha_{1})^{\lambda } \bigr)\,dw \\ &{}+ \frac{f^{(m - 1)}(\alpha_{2}) - f^{(m - 1)}(\alpha_{1})}{(m - 3)!( \alpha_{2} - \alpha_{1})^{2}} \int_{\alpha_{1}}^{\alpha_{2}} \mathsf{K} (\zeta)\,d\zeta + \mathsf{R} _{m}^{1}(\alpha_{1}, \alpha_{2};f),\quad i = 1,2, \end{aligned}$$
  *where the remainder* $\mathsf{R} _{m}^{1}(\alpha_{1},\alpha_{2};f)$ *satisfies the bound*
  33 $$\begin{aligned} \bigl\vert \mathsf{R} _{m}^{1}(\alpha_{1}, \alpha_{2};f) \bigr\vert \le& \frac{1}{\sqrt{2} (m - 3)!} \bigl[\Omega ( \mathsf{K}, \mathsf{K}) \bigr]^{\frac{1}{2}} \\ &{}\times \frac{1}{\sqrt{\alpha_{2} - \alpha_{1}}} \biggl( \int_{\alpha_{1}}^{\alpha_{2}} (\zeta - \alpha _{1}) ( \alpha_{2} - \zeta) \bigl[f^{(m + 1)}(\zeta) \bigr]^{2} \,d\zeta \biggr) ^{ \frac{1}{2}}. \end{aligned}$$
(ii) *For* $G_{2}(z,w)$ *as defined in* (11), *we have*
  34 $$\begin{aligned} \Theta_{i}(f) =& ( - 1) (m - 2) \biggl( \frac{f'(\alpha_{2}) - f'(\alpha _{1})}{\alpha_{2} - \alpha_{1}} \biggr) \int_{\alpha_{1}}^{\alpha_{2}} \Theta_{i} \bigl(G_{2}( \cdot,w) \bigr)\,dw \\ &{}+ \frac{( - 1)}{\alpha_{2} - \alpha _{1}} \int_{\alpha_{1}}^{\alpha_{2}} \Theta_{i} \bigl(G_{2}( \cdot,w) \bigr) \\ &{}\times \sum_{\lambda = 1}^{m - 3} \biggl( \frac{m - 2 - \lambda }{ \lambda!} \biggr) \bigl( f^{(\lambda + 1)}(\alpha_{2}) (w - \alpha_{2})^{ \lambda } - f^{(\lambda + 1)}(\alpha_{1}) (w - \alpha_{1})^{\lambda } \bigr)\,dw \\ &{}+ \frac{f^{(m - 1)}(\alpha_{2}) - f^{(m - 1)}(\alpha_{1})}{(m - 3)!( \alpha_{2} - \alpha_{1})^{2}} \int_{\alpha_{1}}^{\alpha_{2}} \mathsf{K} (\zeta)\,d\zeta + \mathsf{R} _{m}^{2}(\alpha_{1}, \alpha_{2};f), \quad i = 1,2, \end{aligned}$$
  *where the remainder* $\mathsf{R} _{m}^{2}(\alpha_{1},\alpha_{2};f)$ *satisfies the bound*
  $$\begin{aligned} \bigl\vert \mathsf{R} _{m}^{2}(\alpha_{1}, \alpha_{2};f) \bigr\vert \le& \frac{1}{\sqrt{2} (m - 3)!} \bigl[\Omega (\hat{ \mathsf{K} },\hat{\mathsf{K} } ) \bigr]^{\frac{1}{2}} \\ &{}\times \frac{1}{\sqrt{ \alpha_{2} - \alpha_{1}}} \biggl( \int_{\alpha_{1}}^{\alpha_{2}} ( \zeta - \alpha_{1}) ( \alpha_{2} - \zeta) \bigl[f^{(m + 1)}(\zeta) \bigr]^{2} \,d\zeta \biggr) ^{\frac{1}{2}}. \end{aligned}$$

### Proof

(i) Setting $f_{1} \mapsto \mathsf{K} $ and $f_{2} \mapsto f^{(m)}$ in Theorem 3.1, we get

$$\begin{aligned}& \biggl\vert \frac{1}{\alpha_{2} - \alpha_{1}} \int_{\alpha_{1}}^{\alpha_{2}} \mathsf{K} (\zeta)f^{(m)}( \zeta)\,d\zeta - \frac{1}{\alpha_{2} - \alpha _{1}} \int_{\alpha_{1}}^{\alpha_{2}} \mathsf{K} (\zeta)\,d\zeta \cdot \frac{1}{ \alpha_{2} - \alpha_{1}} \int_{\alpha_{1}}^{\alpha_{2}} f^{(m)}(\zeta)\,d\zeta \biggr\vert \\& \quad \le \frac{1}{\sqrt{2}} \bigl[\Omega (\mathsf{K},\mathsf{K}) \bigr]^{\frac{1}{2}} \frac{1}{\sqrt{\alpha_{2} - \alpha_{1}}} \biggl( \int_{\alpha_{1}}^{\alpha_{2}} (\zeta - \alpha_{1}) ( \alpha_{2} - \zeta) \bigl[f^{(m + 1)}(\zeta) \bigr]^{2} \,d\zeta \biggr) ^{\frac{1}{2}}. \end{aligned}$$

Hence, we have

$$\frac{1}{(m - 3)!(\alpha_{2} - \alpha_{1})} \int_{\alpha_{1}}^{\alpha _{2}} \mathsf{K} (\zeta)f^{(m)}\,d \zeta = \frac{f^{(m - 1)}(\alpha_{2}) - f ^{(m - 1)}(\alpha_{1})}{(m - 3)!(\alpha_{2} - \alpha_{1})^{2}} \int_{\alpha_{1}}^{\alpha_{2}} \mathsf{K} (\zeta)\,d\zeta + \mathsf{R} _{m}^{1}( \alpha_{1}, \alpha_{2};f), $$

where the remainder satisfies the estimation (33). Using identity (20) we get (32).

(ii) Similar to the above part. □

The Grüss-type inequalities can be obtained by using Theorem 3.2.

### Theorem 3.4

*Assume* ($H_{1}$), *let* $m \ge 3$ *be an integer*, $f:[\alpha_{1},\alpha_{2}] \to \mathbb{R} $ *be a function such that* $f^{(m)}$ *is absolutely continuous function and* $f^{(m + 1)} \ge 0$ *a*.*e*. *on* $[\alpha _{1},\alpha_{2}]$ *and let the functions* K *and* $\hat{\mathsf{K} } $ *are defined as in* (30) *and* (31). *Then we have*:

(i) *Identity* (32) *where the remainder satisfies the estimation*
  35 $$\begin{aligned} \bigl\vert \mathsf{R} _{m}^{1}(\alpha_{1}, \alpha_{2};f) \bigr\vert \le& \frac{1}{(m - 3)!}\Vert \mathsf{K}'\Vert _{\infty } \\ &{}\times \biggl[ \frac{f^{(m - 1)}(\alpha_{2}) + f^{(m - 1)}( \alpha_{1})}{2} - \frac{f^{(m - 1)}(\alpha_{2}) - f^{(m - 1)}(\alpha _{1})}{\alpha_{2} - \alpha_{1}} \biggr] . \end{aligned}$$
(ii) *Identity* (34) *where the remainder satisfies the estimation*
  36 $$\begin{aligned} \bigl\vert \mathsf{R} _{m}^{1}(\alpha_{1}, \alpha_{2};f) \bigr\vert \le& \frac{1}{(m - 3)!}\Vert \hat{ \mathsf{K}' } \Vert _{\infty } \\ &{}\times \biggl[ \frac{f^{(m - 1)}(\alpha_{2}) + f ^{(m - 1)}(\alpha_{1})}{2} - \frac{f^{(m - 1)}(\alpha_{2}) - f^{(m - 1)}( \alpha_{1})}{\alpha_{2} - \alpha_{1}} \biggr] . \end{aligned}$$

### Proof

(i) Setting $f_{1} \mapsto \mathsf{K} $ and $f_{2} \mapsto f^{(m)}$ in Theorem 3.2, we get

37 $$\begin{aligned}& \biggl\vert \frac{1}{\alpha_{2} - \alpha_{1}} \int_{\alpha_{1}}^{\alpha_{2}} \mathsf{K} (\zeta)f^{(m)}( \zeta)\,d\zeta - \frac{1}{\alpha_{2} - \alpha _{1}} \int_{\alpha_{1}}^{\alpha_{2}} \mathsf{K} (\zeta)\,d\zeta \cdot \frac{1}{ \alpha_{2} - \alpha_{1}} \int_{\alpha_{1}}^{\alpha_{2}} f^{(m)}(\zeta)\,d\zeta \biggr\vert \\& \quad \le \frac{1}{2} \bigl\Vert \mathsf{K} ' \bigr\Vert _{\infty } \frac{1}{\alpha_{2} - \alpha_{1}} \int_{\alpha_{1}}^{\alpha_{2}} (\zeta - \alpha_{1}) ( \alpha_{2} - \zeta) \bigl[f^{(m + 1)}(\zeta) \bigr]^{2} \,d\zeta. \end{aligned}$$

Since

38 $$\begin{aligned}& \int_{\alpha_{1}}^{\alpha_{2}} (\zeta - \alpha_{1}) ( \alpha_{2} - \zeta) \bigl[f^{(m + 1)}(\zeta) \bigr]^{2} \,d\zeta = \int_{\alpha_{1}}^{\alpha _{2}} [2\zeta - \alpha_{1} - \alpha_{2}]f^{m}(\zeta)\,d\zeta \\& \quad = (\alpha_{2} - \alpha_{1}) \bigl[f^{(m - 1)}( \alpha_{2}) + f^{(m - 1)}( \alpha_{1}) \bigr] - 2 \bigl(f^{(m - 1)}(\alpha_{2}) - f^{(m - 1)}( \alpha_{1}) \bigr), \end{aligned}$$

using (20), (37) and (38), we have (35).

(ii) Similar to above part. □

### Theorem 3.5

*Assume* ($H_{1}$), *let* $f:I = [\alpha_{1},\alpha_{2}] \to \mathbb{R} $ *be a function such that* $f^{(m - 1)}$ *is absolutely continuous*, *let* $x_{1}, \ldots,x_{n} \in I$, $p_{1}, \ldots,p_{n}$ *are positive real numbers such that* $\sum_{i = 1}^{n} p_{i} = 1$. *Also*, *let* $F_{\alpha_{1}}^{\alpha_{2}}$, $G_{k}$ ($k = 1,2,3,4$) *and* $\Theta_{i}$ ($i = 1,2$) *are the same as defined in* (8), (10)-(13) *and* (17)-(18), *respectively*. *Moreover*, *assume* $(p,q)$ *is a pair of conjugate exponents that is* $1 \le p,q \le \infty $, $\frac{1}{p} + \frac{1}{q} = 1$. *Let* $\vert f^{(m)}\vert ^{p}:[\alpha_{1},\alpha_{2}] \to \mathbb{R} $ *be a Riemann integrable function*. *Then*:

(i) *For* $k = 1,3,4$, *we have*
  39 $$\begin{aligned}& \Biggl\vert \Theta_{i}(f) - \int_{\alpha_{1}}^{\alpha_{2}} \Biggl[(m - 2) \biggl( \frac{f'( \alpha_{2}) - f'(\alpha_{2})}{\alpha_{2} - \alpha_{1}} \biggr) \\& \qquad {}+ \frac{1}{\alpha_{2} - \alpha_{1}}\sum_{\lambda = 1}^{m - 3} \biggl( \frac{m - 2 - \lambda }{\lambda } \biggr) \\& \qquad {}\times \bigl( f^{(\lambda + 1)}( \alpha_{2}) (w - \alpha_{2}) - f^{(\lambda + 1)}(\alpha_{2}) (w - \alpha _{2}) \bigr) \Biggr]\Theta_{i} \bigl(G_{k}( \cdot,w) \bigr)\,dw \Biggr\vert \\& \quad \le \frac{1}{(\alpha_{2} - \alpha_{1})(m - 3)!} \bigl\Vert f^{(m)} \bigr\Vert _{p} \\& \qquad {}\times \biggl( \int_{\alpha_{1}}^{\alpha_{2}} \biggl\vert \int_{\alpha_{1}}^{\alpha_{2}} \Theta_{i} \bigl(G_{k}( \cdot,w) \bigr) (w - \zeta)^{m - 3}F_{\alpha_{1}}^{\alpha _{2}}( \zeta,w)\,dw \biggr\vert ^{q} \biggr) ^{\frac{1}{q}},\quad i = 1,2. \end{aligned}$$
(ii) *For* $k = 2$, *we have*
  40 $$\begin{aligned}& \Biggl\vert \Theta_{i}(f) - \int_{\alpha_{1}}^{\alpha_{2}} \Biggl[( - 1) (m - 2) \biggl( \frac{f'( \alpha_{2}) - f'(\alpha_{2})}{\alpha_{2} - \alpha_{1}} \biggr) \\& \qquad {}+ \frac{1}{\alpha_{2} - \alpha_{1}}\sum_{\lambda = 1}^{m - 3} \biggl( \frac{m - 2 - \lambda }{\lambda } \biggr) \\& \qquad {}\times \bigl(f^{(\lambda + 1)}(\alpha _{2}) (w - \alpha_{2}) - f^{(\lambda + 1)}(\alpha_{2}) (w - \alpha_{2}) \bigr) \Biggr] \Theta_{i} \bigl(G_{2}( \cdot,w) \bigr)\,dw \Biggr\vert \\& \quad \le \frac{( - 1)}{(\alpha_{2} - \alpha_{1})(m - 3)!} \bigl\Vert f^{(m)} \bigr\Vert _{p} \\& \qquad {}\times \biggl( \int_{\alpha_{1}}^{\alpha_{2}} \biggl\vert \int_{\alpha_{1}}^{\alpha _{2}} \Theta_{i} \bigl(G_{2}( \cdot,w) \bigr) (w - \zeta)^{m - 3}F_{\alpha_{1}} ^{\alpha_{2}}(\zeta,w)\,dw \biggr\vert ^{q} \biggr) ^{\frac{1}{q}},\quad i = 1,2. \end{aligned}$$

### Proof

Similar to the proof of Theorem 3.5 in [8]. □

### Remark 3.6

Similar to Section 4 and Section 5 of [9], the *n*-exponential convexity, mean value theorems and related monotonic Cauchy means (along with examples) can be constructed for the functional defined as the difference between the R.H.S. and the L.H.S. of the generalized inequalities (25) and (26).
